# Flexible Symbiotic Associations of *Symbiodinium* With Five Typical Coral Species in Tropical and Subtropical Reef Regions of the Northern South China Sea

**DOI:** 10.3389/fmicb.2018.02485

**Published:** 2018-11-02

**Authors:** Sanqiang Gong, GuangJun Chai, Yilin Xiao, Lijia Xu, Kefu Yu, Jinlong Li, Fang Liu, Hao Cheng, Fengli Zhang, Baolin Liao, Zhiyong Li

**Affiliations:** ^1^Marine Biotechnology Laboratory, State Key Laboratory of Microbial Metabolism and School of Life Sciences and Biotechnology, Shanghai Jiao Tong University, Shanghai, China; ^2^Coral Reef Research Center of China, Guangxi University, Nanning, China; ^3^Shenzhen Institute of Guangdong Ocean University, Shenzhen, China

**Keywords:** corals, *Symbiodinium*, symbiosis, flexible symbiotic association, tropical, subtropical

## Abstract

The coral symbiont *Symbiodinium* plays important roles in the adaptation of coral to environmental changes. However, coral-*Symbiodinium* symbiotic associations are not well-understood in the South China Sea (SCS) whilst considering environmental factors and host taxa. In this study, next-generation sequencing of the internal transcribed spacer region 2 (ITS2) marker gene was used to explore the symbiotic associations between *Symbiodinium* and five typical coral species across tropical and subtropical reef regions of the SCS. The results showed that *Acropora* sp., *Galaxea fascicularis*, *Platygyra lamellina*, and *Sarcophyton glaucum* exhibited distinct *Symbiodinium* compositions between tropical and subtropical reef regions, whereas *Porites lutea* had stable *Symbiodinium* compositions. More heterogeneous *Symbiodinium* compositions among different coral species were observed in the tropical region, but there were no statistically significant differences in *Symbiodinium* compositions among different coral species in subtropical reef regions. There was a correlation between the *Symbiodinium* compositions and environmental factors, except for the composition of *P. lutea*. *Symbiodinium* subclades D1, D2, C71, C71a, C21, C3b, and C161 were primarily explained by the seawater temperature, nitrate, ammonia, and phosphate. Several host-specific *Symbiodinium* subclades (e.g., C15, C15.6, and C91) were observed in *P. lutea* as well. The findings of this study demonstrate the relationship of *Symbiodinium* diversity with coral hosts and the environment are helpful for elucidating the adaptation of corals to global climate change and anthropogenic disturbance.

## Introduction

Coral symbiont *Symbiodinium*, a photosynthetic algae that is a dinoflagellate, plays a central role in the adaptation of corals and the maintenance of coral reefs (Kemp et al., 2014). Consequently, monitoring *Symbiodinium* compositions and their mutually symbiotic associations with different coral hosts under different environmental stresses is essential to further project the fate of corals and to develop coral reef conservation strategies.

At present, nine *Symbiodinium* clades (known as clades A–I) have been identified (Pochon et al., 2014). Clades A–D are the main *Symbiodinium* harbored by corals, and certain other clades, such as F/G, have also been found in corals (Baker, 2003). Within any given clade, different *Symbiodinium* subclades are mainly identified by the commonly used polymerase chain reaction-denaturing gradient gel electrophoresis (PCR-DGGE) profiles of the less-conserved *Symbiodinium* internal transcribed spacer region 2 (ITS2) sequence (LaJeunesse et al., 2003). Previous studies have revealed that the environment could influence *Symbiodinium* compositions, as well as their mutually symbiotic associations with corals, and the breakdown of *Symbiodinium*-coral associations because of the effects of climate change and anthropogenic disturbance that would contribute to dramatic declines in coral reef cover worldwide (Hughes et al., 2003, 2017; Hoegh-Guldberg et al., 2007). In contrast, certain coral species still maintain highly stable coral-*Symbiodinium* symbiotic associations over broad geographical ranges and temperature gradients, as well as through bleaching events or after transplantation (LaJeunesse et al., 2004a,b; Stat et al., 2009; Thomas et al., 2014). Therefore, corals might adapt to environmental changes via different mechanisms (Warner et al., 2006). For example, most corals can adapt to environmental changes by changing their symbiont communities, but some corals (most notably the genus *Porites*) remain common on devastated reefs, even though they fail to change their symbiont communities.

The South China Sea (SCS) has extensive an coral reef development (at least 8,000 km^2^, including at least 571 coral species), and coral reefs mainly develop in the Nansha Islands, Zhongsha Islands, Xisha Islands, Hainan Island, Leizhou Peninsula, and Dongsha Islands (Yu, 2012; Huang et al., 2015). The *Symbiodinium* subclades associated with coral species in the Nansha Islands (Huang et al., 2006), Xisha Islands (Dong et al., 2009), Dongsha Islands (Dong et al., 2008a), Hainan Island (specifically the Luhuitou fringing reef, Dong et al., 2008b), and Taiwan Island (Chen et al., 2005a,b; Keshavmurthy et al., 2014, 2017) have mainly been investigated using DGGE-based analyses. However, the DGGE-based method is unable to detect *Symbiodinium* subclades with relative abundances of less than 10% and, thus, may lack sufficient sensitivity to detect the change in symbiosis between corals and *Symbiodinium* (Mieog et al., 2007). The next-generation sequencing of *Symbiodinium* ITS2 region offers a highly sensitive method that is able to detect low-abundance *Symbiodinium* subclades and is reliable enough for measurements of *Symbiodinium* subclade compositions in corals (Arif et al., 2014; Thomas et al., 2014; Boulotte et al., 2016; Cunning et al., 2017). Most recently, Tong et al. (2017) analyzed *Symbiodinium* subclade compositions in *Galaxea fascicularis* (scleractinian) and *Montipora* spp. (scleractinian) in tropical (Hainan Island and Xisha Island) and subtropical (Hong Kong) reef regions of the SCS by next-generation sequencing of *Symbiodinium* ITS2 region, suggesting that temperature is the main factor that drives coral-*Symbiodinium* symbiotic associations. A similar result was also reported by Zhou et al. (2017) in *G. fascicularis* in the SCS. However, knowledge gaps still remain. At present, little is known regarding the diversity of *Symbiodinium* and their symbiotic associations with abundant corals (such as *Acropora* and *Porites*) in the SCS whilst considering environmental factors and host taxa.

In this study, the dominant coral species of *Acropora* sp. (scleractinian) and *Porites lutea* (scleractinian) as well as the three common coral species of *G. fascicularis*, *Platygyra lamellina* (scleractinian), and *Sarcophyton glaucum* (alcyonacea) were sampled from different reef sites across tropical (Sanya Bay-SY, Hainan Island) and subtropical (Xuwen County-XW, Leizhou Peninsula, and/or Daya Bay-DY) reef regions of the SCS. The *Symbiodinium* compositions in these coral samples in the SY, XW, and DY reef regions were analyzed by next-generation sequencing of *Symbiodinium* ITS2 region, aiming to elucidate coral-*Symbiodinium* symbiotic associations across these regions. Furthermore, the relationship of *Symbiodinium* compositions with environmental factors and host taxa were analyzed.

## Materials and Methods

### Sampling Regions and Sample Collection

**FIGURE 1:**
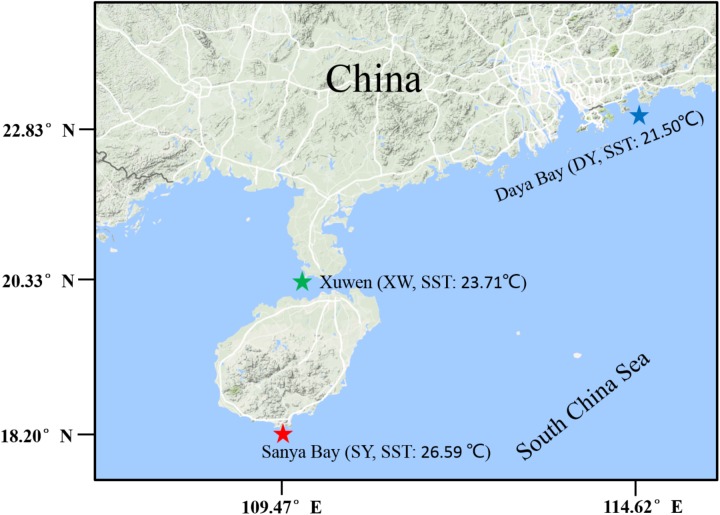
Sampling locations. Sampling regions are indicated by stars. The red star represents the tropical reef region of Sanya Bay (SY), the green star represents the subtropical reef region of Xuwen (XW) County, and the blue star represents the subtropical reef region of Daya Bay (DY).

Sampling regions of SY (E109.470°-109.489°, N18.200°-18.217°), XW (E109.867°-109.869°, N20.333°-20.332°), and DY (E114.621°-114.611°, N22.833°-22.762°) are located in the northern SCS (Figure 1). The SY reef region, Hainan Island, China has a typical tropical ocean climate, and the mean annual surface seawater temperature (SST) in 2015 was 26.59°C (Supplementary Table 1); this region has been listed as part of the National Coral Reef Natural Reserves since 1990 (Li et al., 2008). The XW reef region, Leizhou Peninsula, is located on the northern coast of the SCS with a subtropical climate (Yu, 2000), and the mean annual SST of XW reef region in 2015 was 23.71°C (Supplementary Table 1). The XW reef region has been listed as part of the National Coral Reef Natural Reserves since 2007. The DY reef region is located to the southeast of Shenzhen City of Guangdong Province, China and has a typical subtropical ocean climate (Chen et al., 2009), and the mean annual SST in this region in 2015 was 21.50°C (Supplementary Table 1).

Coral samples of *Acropora* sp., *G. fascicularis*, *P. lamellina*, *P. lutea*, and *S. glaucum* were obtained from the SY, XW, and DY reef regions in August and September 2015 (Table 1). These coral samples were identified based on the morphology and a molecular barcode analysis of the *cytb* gene. The *cytb* gene was amplified by PCR with previously reported primers for corals (Fukami et al., 2004), and it was cloned into the pEASY’T5 Zero Cloning Vector (Transgene Biotech, Beijing, China). Clone per coral sample was picked up for further Sanger sequencing. An alignment analysis of *cytb* gene was performed by BLASTN against the NCBI database^1^. Coral samples with *cytb* gene sequence similarity ≥98% and with similar morphology were identified as the same coral species. The representative sequences of *cytb* genes obtained in this study have been submitted to the NCBI database with accession numbers MH160422–MH160426 (Supplementary Data Sheet 1). At each reef region, coral colonies from different reef sites (at least three reef sites) separated by at least 100 m were collected. Fragments of approximately 1 cm^2^ were picked and washed with filtered seawater (0.22-μm polycarbonate membranes) at least three times.

**Table 1:** Coral samples from SY, XW, and DY reef regions of the northern SCS, including sampling regions (latitudes and longitudes), climate, and coral samples information.

Regions	Climate	Coral	Sample size (*n*)^a^	Date (yyyy-mm-dd)
Sanya Bay (SY) E109.470°-109.489° N18.200°-18.217°	Tropical	*Acropora* sp.	8	2015-08-24
		*G. fascicularis*	5	2015-08-25
		*P. lamellina*	5	2015-08-25
		*P. lutea*	4	2015-08-26
		*S. glaucum*	4	2015-08-25
Xuwen (XW) E109.867°-109.869° N20.333°-20.332°	Subtropical	*Acropora* sp.	3	2015-08-29
		*G. fascicularis*	4	2015-08-29
		*P. lamellina*	5	2015-08-29
		*P. lutea*	5	2015-08-29
		*S. glaucum*	5	2015-08-29
Daya Bay (DY) E114.621°-114.611° N22.833°-22.762°	Subtropical	*Acropora* sp.	10	2015-09-02
		*G. fascicularis*	10	2015-09-02
		*P. lamellina*	4	2015-09-02
		*P. lutea*	5	2015-09-02

*^a^The number of coral samples from different coral sites.*

The seawater samples from at least five reef sites in each reef region were collected and filtered using 0.22-μm polycarbonate membranes (Whatman GF/F, 47 mm). Next, the filtered seawater from each of the different reef sites, that is, SY, XW, and DY reef regions, were mixed. All samples were preserved in RNAlater^®^ at 4°C in the field and stored at -80°C in the lab until DNA extraction was conducted.

### Environmental Data Collection

The mean annual SSTs of the SY, XW, and DY reef regions were calculated based on the SST of these reef regions in spring, summer, autumn, and winter of 2015 (Supplementary Table 1).

The SST, salinity, and depth of different reef regions (at least five reef sites in each reef region) were measured by OCEAN SEVEN 320 Plus CTD (Idronaut, Italy). Dissolved oxygen (DO) was detected using a YSI 6600V2-02 multi-parameter instrument (YSI, United States). Dissolved nutrients (nitrite-NO_2_^-^, nitrate-NO_3_^-^, ammonia-NH_4_^+^, and phosphate-PO_4_^3-^) were analyzed using a Lachat QC8500 Flow Injection Autoanalyzer (Lachat Instruments, United States) (Supplementary Table 2). The differences in environmental factors among different coral reef regions were tested using a multiple comparison test (Tukey’s HSD) in the R software environment (R 3.1.2).

### DNA Extraction

Total DNA of each coral sample was extracted using the Qiagen DNeasy Plant Mini Kit (Qiagen, Hilden, Germany) according to the manufacturer’s protocol. The integrity of the DNA was monitored by 0.8% (w/v) agarose gel electrophoresis stained with 1 × SYBR Safe (Invitrogen, Carlsbad, United States). The amount of DNA was determined by a Qubit^®^ 3.0 Fluorometer (Life Technologies, United States). The purity of the DNA was determined by NanoDrop^TM^ 2000 Spectrophotometer (Thermo Scientific, United States). All DNA samples were stored at -20°C for further use.

### Amplicon Sequencing

The ITS2 region of the ribosomal RNA gene of *Symbiodinium* was PCR amplified using a pair of barcoded primers: ITSintfor2 (5′-GAATTGCAGAACTCCGTG-3′) and ITS2-reverse (5′-GGGATCCATATGCTTAAGTTCAGCGGGT-3′) (LaJeunesse and Trench, 2000). The PCR was performed with 12.5 μl of a Qiagen Multiplex PCR reagent (Qiagen, Hilden, Germany), 0.1 μM primer, 50 ng of DNA, and DNase-free water to make a total volume of 25 μl. The following PCR conditions were used: initial denaturation for 3 min at 94°C, followed by 34 cycles of 98°C for 10 s, 51°C for 30 s, 68°C for 30 s, and a final extension step of 5 min at 68°C. The DNA libraries were validated using an Agilent 2100 Bioanalyzer (Agilent Technologies, Palo Alto, CA, United States) and were quantified using a Qubit^®^ 3.0 Fluorometer (Life Technologies, United States). All qualified amplification products were mixed in equal amounts followed by sequencing on an Illumina MiSeq instrument (Illumina, San Diego, CA, United States) according to the manufacturer’s instructions using a 300 × 2 paired-end configuration. The raw data were submitted to the NCBI Sequence Read Archive (SUB2393447).

### Data Processing and Data Analysis

According to the methods and script supplied by Cunning et al. (2017), paired reads from each sample were merged using the illumina-utils software (Eren et al., 2013) with an enforced Q30-check. Only sequences with overlap ≥150 bp and mismatch ≤3 bp were retained. Chimeric sequences were removed using USEARCH 6.1 (Edgar, 2010) implemented in QIIME (Caporaso et al., 2010). Primers were trimmed using cutadapt (Martin, 2011), allowing three mismatches. Only sequences with both forward and reverse primer matches and length ≥250 and ≤380 bp after trimming were retained. Qualified ITS2 sequences were clustered into operational taxonomic units (OTUs) at 97% identity within samples (e.g., sequences from each sample clustered independently). The OTUs that represented ≤3 ITS2 sequences were removed. The most abundant sequence from each OTU was chosen as the representative sequence and classified as a *Symbiodinium* subclade against a custom reference database of *Symbiodinium* subclades (Tong et al., 2017) using the Needleman-Wunsch global alignment algorithm implemented in the Biostrings package (Pagès et al., 2016) in R (R Core Team, 2014). Each OTU was then assigned a *Symbiodinium* subclade name with the highest alignment score.

The resulting *Symbiodinium* subclades count tables of different samples were merged for downstream statistical analysis. The Shannon–Wiener (H′) diversity index was calculated in the R software environment (R 3.1.2) to assess the alpha-diversity of *Symbiodinium* subclades associated with coral samples. The differences in the Shannon–Wiener (H′) diversity index of *Symbiodinium* subclades among different samples was tested with the multiple comparison test (Tukey’s HSD) using R software (R 3.1.2). A rarefaction curve was plotted using “make_rarefaction_plots.py” script in the QIIME package based on the merged count table (Caporaso et al., 2010). To present the relationships of the *Symbiodinium* subclade compositions in different coral samples from different reef regions, a Bray–Curtis dissimilarity-based principal coordinates analysis (PCoA) of *Symbiodinium* subclade compositions was performed by the vegan package in R (Oksanen et al., 2016). To test the relationships among environmental factors, *Symbiodinium* subclade compositions, and reef regions, a canonical correspondence analysis (CCA) was conducted in R using the vegan package (Oksanen et al., 2016).

To identify the phylogenetic relationships of *Symbiodinium* subclades with coral hosts, phylogenetic trees were constructed using two different methods: the Bayesian inference (BI) and maximum likelihood (ML) methods. Analyses were performed with the Kimura 2-parameter model, and the evolutionary models that fit best for the datasets were calculated using the program JModelTest version 2.1.7 (Darriba et al., 2012). For BI, the chain was run for 10 million generations, saving every 1,000th generation in Mrbayes (Ronquist et al., 2012). For ML, we performed 1,000 replicates of the rapid bootstrapping algorithm in MEGA version 6.06 (Tamura et al., 2013). Nearly identical trees were obtained; thus, only the ML tree was presented in this study.

## Results

### Overall Diversity of *Symbiodinium* Subclades in Explored Corals in SY, XW, and DY Reef Regions

For 77 coral samples, a total of 4,458,888 *Symbiodinium* ITS2 sequences (average 57,907 *Symbiodinium* ITS2 sequences per coral sample) were retained after quality control. Thirty-two *Symbiodinium* subclades were classified (Supplementary Table 3 and Supplementary File 1), of which 13 *Symbiodinium* subclades (covering more than 95% of the total *Symbiodinium* ITS2 sequences) were selected for further analysis, while the rest (19 *Symbiodinium* subclades) were designated as others (Supplementary File 2).

For each coral sample, rarefaction curves (Supplementary Figure 1) showed that the number of observed *Symbiodinium* subclades reached an asymptote, indicating that the number of *Symbiodinium* ITS2 sequences from each coral sample was able to meet current needs for the diversity analysis of *Symbiodinium* subclades.

**FIGURE 2:**
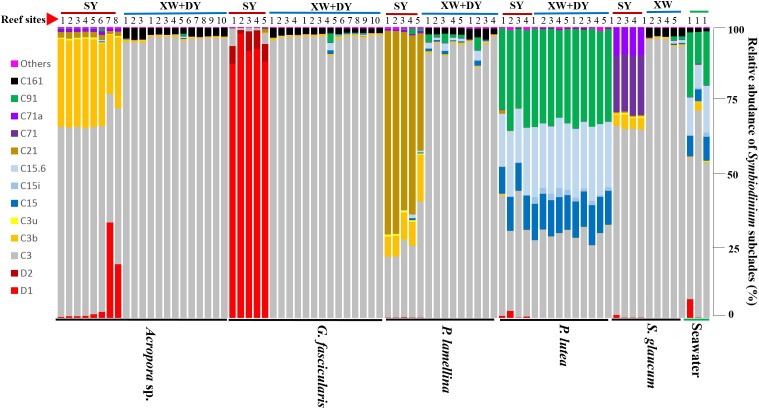
Bar plot of the relative abundance of different *Symbiodinium* subclades in coral samples and filters of seawaters from tropical (red color code) and subtropical (blue color code) reefs in the northern SCS. Each bar represents the relative abundance of different *Symbiodinium* subclades in one sample.

It was noted that *Acropora* sp., *G. fascicularis*, *P. lamellina*, and *S. glaucum* exhibited distinct *Symbiodinium* subclade compositions between tropical and subtropical reef regions, whereas *P. lutea* failed to exhibit differences in *Symbiodinium* subclade compositions between tropical and subtropical reef regions (Figure 2). *Symbiodinium* subclade compositions among different coral species were more heterogeneous in the SY reef region, but there were no notable differences in *Symbiodinium* subclade compositions among the explored coral species in the XW and DY reef regions. Specifically, the *Symbiodinium* subclade C3 was dominant in most explored corals, the subclade D1 was mainly detected in corals in the SY reef region, and the subclade C161 was mainly associated with corals in the XW and DY reef regions. In addition, nearly all *Symbiodinium* subclades observed in explored coral samples were also detected in seawater samples, implying a possible linkage between *Symbiodinium* subclades in coral hosts and those in seawater.

**FIGURE 3:**
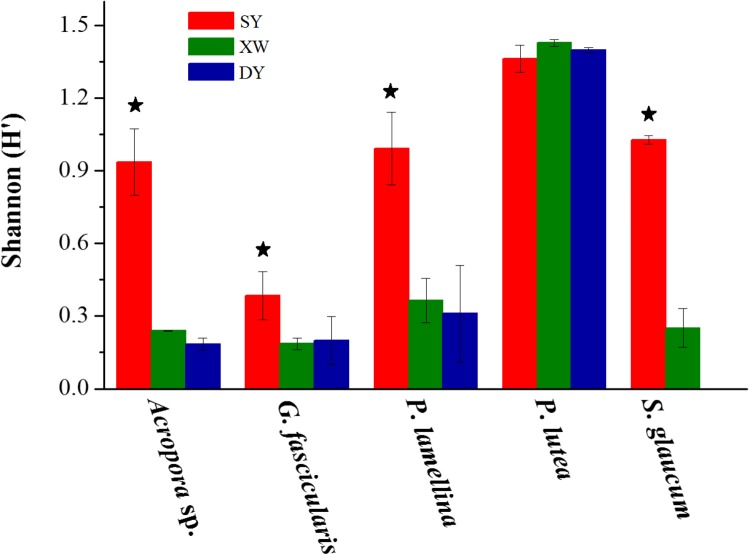
Bar plot of the Shannon (H′) diversity index values of *Symbiodinium* subclades in the same coral species or genus living in tropical SY and subtropical XW and DY reef regions in the northern SCS. The Shannon (H′) diversity index values are mean ± SE. Stars above the bar plots denote statistically significant differences between the same coral species or genus living in tropical SY and subtropical XW or DY (*P* < 0.001).

The same coral species or genus in the SY reef region showed higher Shannon–Wiener (H′) index values of *Symbiodinium* subclades than corresponding corals in the XW or DY reef regions (*P* < 0.001) (Figure 3). One exception was *P. lutea*, as its Shannon–Wiener (H′) index values of *Symbiodinium* subclades were not statistically significantly different across the three reef regions (*P* > 0.05).

### Relationship of *Symbiodinium* Subclade Compositions With Environmental Factors and Hosts

**FIGURE 4:**
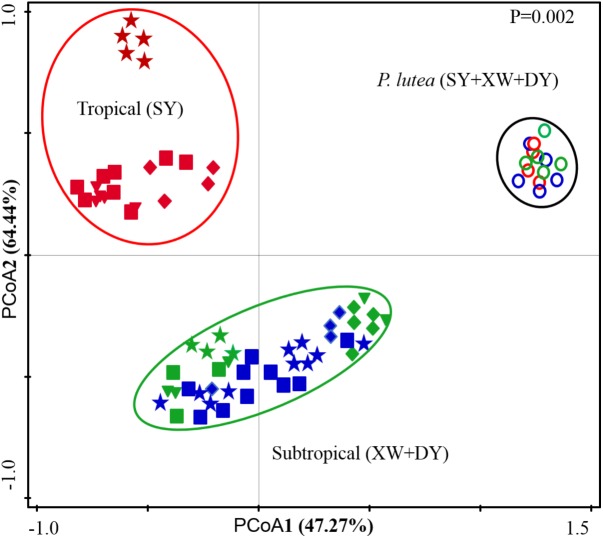
Principle coordinates analysis (PCoA) scores plot based on read counts of *Symbiodinium* subclades in coral samples from the tropical SY and subtropical XW and DY reefs in the northern SCS. The square (■) represents *Acropora* sp.; the star (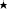) represents *G. fascicularis*; the diamond (♦) represents *P. lamellina*; the circle (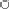) represents *P. lutea*; and the triangle (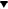) represents *S. glaucum*. For the same coral species or genus samples, different colors represent different reefs (red represents coral samples from tropical SY; green represents coral samples from subtropical XW; and blue represents coral samples from subtropical DY).

A PCoA based on *Symbiodinium* subclade counts of different coral samples in the SY, XW, and DY reef regions revealed a segregation of *Symbiodinium* subclade compositions (Figure 4). The *Symbiodinium* subclade compositions of *Acropora* sp., *G. fascicularis*, *P. lamellina*, and *S. glaucum* in the SY region were grouped together, whereas the *Symbiodinium* subclade compositions of *Acropora* sp., *G. fascicularis*, *P. lamellina*, and *S. glaucum* in the XW and DY reef regions were grouped together. However, the *Symbiodinium* subclade compositions of *P. lutea* in the SY, XW, and DY reef regions were grouped together. The Monte Carlo permutation test showed that the *Symbiodinium* subclade compositions among these three groups were significantly different (*P* = 0.002).

**FIGURE 5:**
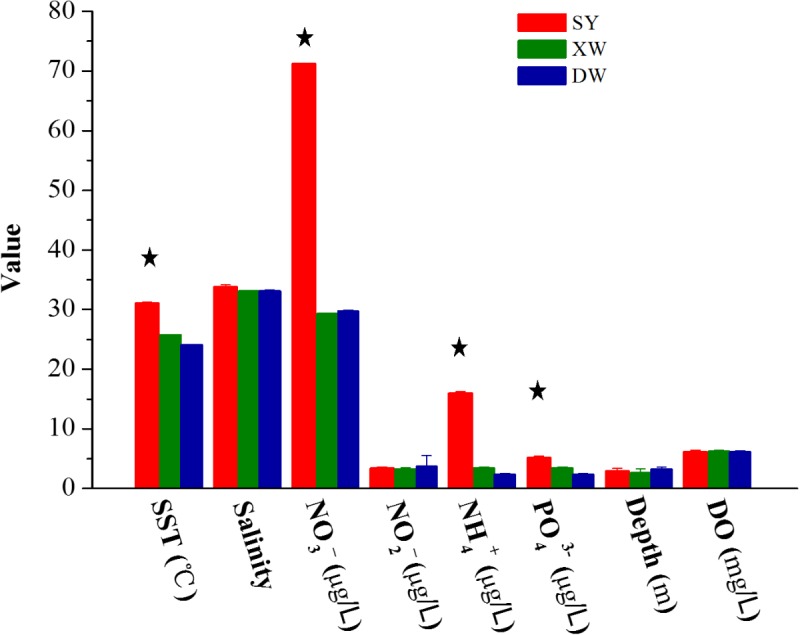
Bar plot of the value of environmental factors in the tropical SY and subtropical XW and DY reef regions in the northern SCS. The value of environmental factors is mean ± SE (SY, *n* = 8; XW, *n* = 5; DY, *n* = 10). Stars above bar plots denote statistically significant differences between tropical SY and subtropical XW or DY (*P* < 0.001).

**FIGURE 6:**
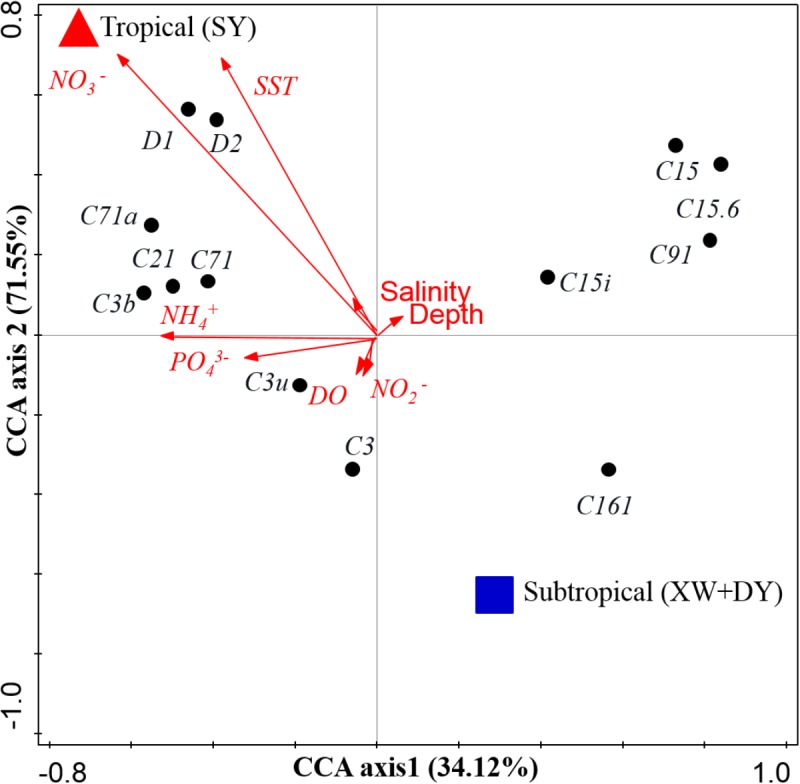
Biplot of the canonical correspondence analysis (CCA) of *Symbiodinium* subclades with environmental factors. Red triangles represent the tropical SY reef region, and blue squares represent the subtropical DY and XW reef regions.

The distinct *Symbiodinium* subclade compositions in explored corals living between tropical (SY) and subtropical (XW and DY) reef regions, except those in *P. lutea*, were probably explained by the different environmental factors of the different reef regions (Figures 5, 6). According to the CCA (Figure 6), the SST and NO_3_^-^, NH_4_^+^, and PO_4_^3-^ served as the main factors affecting *Symbiodinium* subclade compositions in tropical (SY) and subtropical (XW and DY) reef regions. The *Symbiodinium* subclades D1, D2, C71, C71a, C21, and C3b mainly occurred in the SY reef region and were positively correlated with the SST and NO_3_^-^, NH_4_^+^, and PO_4_^3-^. Conversely, the *Symbiodinium* subclade C161 occurred predominantly in the XW and DY reef regions and was negatively correlated with those environmental factors. However, there was no clear correlation between *Symbiodinium* subclades C15, C15.6, C91, and C15i (mainly associated with *P. lutea*) and all of the measured environmental factors.

### Phylogenetic Relationship of *Symbiodinium* Subclades From Explored Corals

**FIGURE 7:**
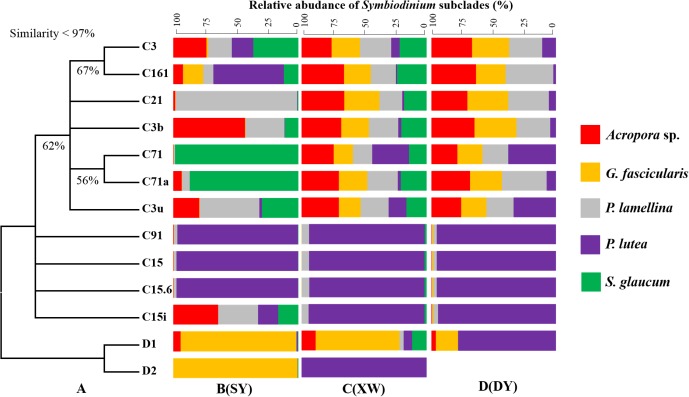
Combination chart of the phylogenetic tree **(A)** and bar plot of *Symbiodinium* subclades in explored corals living in the SY **(B)**, XW **(C)**, and DY **(D)** reef regions. The phylogenetic tree was drawn based on ITS2 sequences of different *Symbiodinium* subclades using the maximum likelihood and neighbor joining methods, and nearly identical trees were obtained. The presented tree is derived from the maximum likelihood method (bootstrap values below 50% are not shown). The bar plot was drawn based on the ITS2 sequences of *Symbiodinium* subclades in explored corals. Each bar represents the relative abundance (mean) of one *Symbiodinium* subclade in different coral species.

The similarities of ITS2 sequences of different *Symbiodinium* subclades used for phylogenetic tree construction were less than 97%, supporting the fact that the 13 *Symbiodinium* subclades from explored coral samples were distinct (Supplementary Data Sheet 2). The bootstrap values of correlated *Symbiodinium* subclades were less than 70% in the ML phylogenetic tree (Figure 7A), revealing low confidence to support branches among those *Symbiodinium* subclades. Based on the 13 *Symbiodinium* subclade compositions detected in different coral species in SY, XW, and DY reef regions, it was noted that different coral species could be associated with multiple *Symbiodinium* subclades and vice versa, suggesting flexible coral-*Symbiodinium* symbiotic associations. Interestingly, the *Symbiodinium* subclades C15, C15.6, and C91 seemed to be specific to *P. lutea*. The three subclades were the most dominant *Symbiodinium* in *P. lutea*, across tropical and subtropical reef regions, and they showed stable compositions (Figure 7).

## Discussion

In this study, we investigated *Symbiodinium* subclade compositions in five typical coral species across the SY, XW, and DY reef regions in the northern SCS. Several studies have investigated *Symbiodinium* subclade compositions of certain corals in the SCS and suggested that temperature (SST) could shape coral-*Symbiodinium* symbiotic associations (Zhou and Huang, 2011; Tong et al., 2017; Zhou et al., 2017). According to this study, in addition to SST, other environmental factors, especially NO_3_^-^, NH_4_^+^, and PO_4_^3-^, and host taxa affect coral-*Symbiodinium* symbiotic associations in the SCS as well.

### Environmental Factors Affect the Coral-*Symbiodinium* Symbiotic Associations

It has been suggested that corals may adapt to environmental changes by regulating their hosted *Symbiodinium* (Cunning et al., 2015a,b). For example, Keshavmurthy et al. (2014) explored corals exposed to long-term elevated SST in the presence of a nuclear power plant outlet and found that corals changed their symbiotic *Symbiodinium* subclades from C1 and C3 (stress sensitive) to D1a (stress tolerant) or to a mixture of *Symbiodinium* subclades C1/C3/C21a/C15 and D1a. Corals from some extremely hot regions with high SST were found to host heat tolerant *Symbiodinium*, that is, subclades within clade D (Berkelmans and van Oppen, 2006; Lajeunesse et al., 2009; D’Angelo et al., 2015). Extremely high temperatures could lead to coral bleaching, but corals that hosted more heat tolerant *Symbiodinium* were less likely to bleach; several bleached corals gained more heat tolerant *Symbiodinium* after recovery (Kemp et al., 2014). The recent study by Tong et al. (2017) about *G. fascicularis* and *Montipora* spp. in the SCS showed that the putative heat tolerant *Symbiodinium* subclades within clade D were mainly detected in corals across tropical reef regions, and *Symbiodinium* subclade compositions in explored corals were distinct between tropical and subtropical reef regions, suggesting that the SST mainly shapes coral–algal symbiosis. Zhou et al. (2017) found that SST shaped *G. fascicularis*-algal symbiosis in the SCS. Based on this study, in addition to SST, other environmental factors, such as seawater NO_3_^-^, NH_4_^+^, and PO_4_^3-^, and host taxa could also affect the coral-*Symbiodinium* symbiotic associations in the SCS. The compositions of *Symbiodinium* subclades in *Acropora* sp., *G. fascicularis*, *P. lamellina*, and *S. glaucum* from the tropical reef region (SY) with high SST, NO_3_^-^, NH_4_^+^, and PO_4_^3-^ were distinct from those from the subtropical reef regions (XW + DY), suggesting that nutrient inflow might also affect coral-*Symbiodinium* symbiotic associations in addition to SST. These results are consistent with Sawall et al. (2014), who suggested that nutrients have the potential to affect *Symbiodinium* community structures associated with *Pocillopora verrucosa* in the Red Sea.

### Host Affects the Coral-*Symbiodinium* Symbiotic Associations

In the Indo-Pacific Ocean, previous studies have shown that *Porites*-*Symbiodinium* symbiotic associations were stable over broad geographical scales and temperature ranges as well as through bleaching events or after transplantation (LaJeunesse et al., 2004a,b; Stat et al., 2009). Similar to these studies, our present results based on high-throughput sequencing show that the *Symbiodinium* subclade compositions of *P. lutea* (*Porites*) are not statistically and significantly different across subtropical and tropical reef regions in the SCS. In addition, there are no clear correlations of the *Symbiodinium* subclades C15, C15.6, and C91 mainly detected in *P. lutea* with all detected environmental factors. All of these results reveal that the host could also affect *Symbiodinium* subclade compositions, and the *Symbiodinium* subclades C15, C15.6, and C91 seem to be specific to *P. lutea*.

### Ecological Implications

The adaptation of corals to future climate change and anthropogenic disturbances strongly relies on their hosted *Symbiodinium*. In the SCS, temperature is one of the measured environmental factors that could shape coral-*Symbiodinium* symbiotic associations. Therefore, the rise of SST is a potential threat to corals in the SCS. Previous studies have shown that heat-tolerant corals contain a greater number of clade D *Symbiodinium*. Similar to Tong et al. (2017), the present results also show that *G. fascicularis* in the SCS appears to host more heat-tolerant *Symbiodinium* subclades within clade D, which may lead to their dominance during future climate change. In addition, the present study shows that other environmental factors (especially NO_3_^-^, NH_4_^+^, and PO_4_^3-^) can also serve as major factors affecting coral-*Symbiodinium* symbiotic associations. Increases in coastal nutrients are frequently linked to human activities (Doney, 2010), which suggests that human activities may also affect coral-*Symbiodinium* symbiotic associations. The present results suggest that seawater pollution may be another potential threat to the explored corals in the SCS. A “nugget of hope” for these threats is that most explored corals in this study have the potential to adapt to future climate change with the flexibility of their symbiosis with *Symbiodinium*. One exception is the coral *P. lutea*. The present results show that *Symbiodinium* compositions in *P. lutea* are probably affected by host itself, and the *Symbiodinium* subclades C15, C15.6, and C91 seem to be specific to *P. lutea*. According to Warner et al. (2006), this type of specificity favors the evolution of mutualism in symbiosis, thereby, increasing the persistence of intimate and long-term mutualisms. In fact, in recent years, certain dominant coral species have been declining, while certain subdominant coral species are becoming more dominant (Grottoli et al., 2014). For example, the coral community structure in SY (Luhuitou, China) changed dramatically into the current predominance of massive *Porites* (primarily *P. lutea*) (Zhao et al., 2016). Based on the above results, the specificity between *Symbiodinium* and *P. lutea* may be a reason for *P. lutea* being the dominant coral in the SCS.

## Conclusion

This study demonstrates the multiple relationships of the *Symbiodinium* community with both the coral host and the environment. Corals might adapt to different environmental conditions by changing their *Symbiodinium* communities (e.g., taking up the more heat-tolerant clade D *Symbiodinium*). Conversely, certain corals, such as *P. lutea*, might be able to adapt to different environmental conditions (e.g., climate change and elevated nutrient inflow) via host-specific *Symbiodinium* subclades (e.g., C15, C15.6, and C91). Elucidating detailed mechanisms of corals’ adaptation to different environments warrants future investigation in both field and lab settings.

## Data Accessibility

The raw sequence information in this paper has been deposited in the GenBank Sequence Read Archive with accession number SUB2393447.

## Author Contributions

SG and ZL conceived and designed the experiments. SG performed the experiments and analyzed the experimental data. SG, LX, JL, FL, HC, and BL contributed to reagents and materials. SG, ZL, GC, YX, FZ, and KY wrote the manuscript.

## Conflict of Interest Statement

The authors declare that the research was conducted in the absence of any commercial or financial relationships that could be construed as a potential conflict of interest.The reviewer CA-G and handling Editor declared their shared affiliation at time of review.

## Footnotes

1 https://blast.ncbi.nlm.nih.gov/
