# Supplementary material for: Flexible Symbiotic Associations of Symbiodinium With Five Typical Coral Species in Tropical and Subtropical Reef Regions of the Northern South China Sea
Source: Front Microbiol. 2018 Nov 2;9:2485. doi: 10.3389/fmicb.2018.02485 (PMC6225575; doi:10.3389/fmicb.2018.02485)
Supplement: Supplementary file 4 [file Presentation_1.pdf]

Supplementary Data for:

Flexibly symbiotic associations of *Symbiodinium* with five typical coral species in tropical and subtropical reef regions of the northern South China Sea

Sanqiang Gong[1], GuangJun Chai[1], Yilin Xiao[1], Lijia Xu[2], Kefu Yu[2], Jinlong Li[1], Fang Liu[1], Hao Cheng[1], Fengli Zhang[1], Baolin Liao[3], Zhiyong Li[1,]*

[1]Marine Biotechnology Laboratory, State Key Laboratory of Microbial Metabolism and School of Life Sciences & Biotechnology, Shanghai Jiao Tong University, 800 Dongchuan Road, Shanghai 200240, Peoples Republic of China
[2]Coral Reef Research Center of China, Guangxi University, Nanning 530004, China
[3]Shenzhen Institute of Guangdong Ocean University, Shenzhen 518108, China

**Corresponding author: Zhiyong Li*
Tel.: +86-21-34204036; fax: +86-21-34204036

E-mail: zyli@sjtu.edu.cn

Contents:

Supplementary Table 1. The mean annual sea surface temperature (SST, °C) for different sampling reef regions (2015, mean ± sd).

Reef regions	Spring	Summer	Autumn	Winter	Mean SST
Sanya (SY)	25.28±0.79	30.46±1.52	27.34±0.57	23.28±0.46	26.59 ± 3.06
Xuwen (XW)	21.78±0.54	28.32±0.61	24.69±0.60	20.03±0.82	23.71 ±3.63
Daya Bay (DY)	19.11±0.49	26.14±0.83	22.74±0.41	18.02±0.76	21.50 ±3.69

Supplementary Table 2. Environmental data during sampling period (mean ± sd).

Reef regions	Temperature (°C)	Depth (m)	Salinity	DO (mg/L)	NO_3^- (μg/L)	NO_2^- (μg/L)	NH_4^+ (μg/L)	PO_4^{3-} (μg/L)
Sanya (SY) E109.470°-109.489° N18.200°-18.217°	31.12 ± 0.70	2.94 ± 0.45	33.82 ± 0.34	6.14 ± 0.31	71.23 ± 0.10	3.42 ± 0.22	16.02 ± 0.33	5.23 ± 0.18 ±
Xuwen (XW) E109.867°-109.869° N20.333°-20.332°	25.78 ± 0.42	2.64 ± 0.66	33.12 ± 0.02	6.23 ± 0.22	29.33 ± 0.10	3.28 ± 0.23	10.12 ± 0.22	3.48 ± 0.12
Daya Bay (DY) E114.621°-114.611° N22.833°-22.762°	24.11± 0.62	3.22 ± 0.33	33.12 ± 0.13	6.16 ± 0.17	29.77 ± 0.17	3.75 ± 1.75	10.33 ± 0.16	2.37 ± 0.15

Supplementary Table 3. Summary of NGS data and Shannon diversity index. Including sampled corals, number of ITS2 sequences post quality control (QC), number of *Symbiodinium* ITS2 sequences, number of *Symbiodinium* subclades and Simpson diversity indices.

**Corals**	**QC sequences**	**Number of *Symbiodinium* ITS2 sequences**	**Number of *Symbiodinium* subclades (> 0.1%)**	**Shannon (H')**
A. sp. SY1	52421	52417	16(12)	0.8245
A. sp. SY2	44335	44330	15(12)	0.853
A. sp. SY3	47226	47223	15(12)	0.8396
A. sp. SY4	45675	45673	14(11)	0.8381
A. sp. SY5	52743	52717	17(12)	0.8791
A. sp. SY6	42262	42174	21(13)	0.9611
A. sp. SY7	44872	44867	16(12)	1.16
A. sp. SY8	46767	46767	16(10)	1.135

A. sp. XW1	51092	50922	13(11)	0.2396
A. sp. XW2	63689	63598	13(9)	0.2375
A. sp. XW3	73507	73393	15(11)	0.2397
A. sp. DY1	61270	61155	16(11)	0.1676
A. sp. DY2	50414	50401	14(11)	0.1667
A. sp. DY3	64193	63749	14(11)	0.1694
A. sp. DY4	81109	76406	13(10)	0.1664
A. sp. DY5	41681	41644	17(12)	0.2506
A. sp. DY6	49127	48987	15(11)	0.1916
A. sp. DY7	44404	44361	13(10)	0.1876
A. sp. DY8	76787	76551	17(11)	0.1833
A. sp. DY9	80037	78426	17(11)	0.1792
A. sp. DY10	49394	49270	12(9)	0.1773
G. fascicularis SY1	55305	55173	14(11)	0.4746
G. fascicularis SY2	68083	67969	15(12)	0.3211
G. fascicularis SY3	56814	55660	13(11)	0.3236
G. fascicularis SY4	41063	40942	15(11)	0.296
G. fascicularis SY5	56041	54521	20(12)	0.5093
G. fascicularis XW1	57972	57972	15(11)	0.2195
G. fascicularis XW2	49977	49977	14(11)	0.1843
G. fascicularis XW3	60283	60280	14(11)	0.1671
G. fascicularis XW4	64397	64377	14(11)	0.1701
G. fascicularis DY1	47388	47386	14(11)	0.1636
G. fascicularis DY2	56865	56864	13(9)	0.1609
G. fascicularis DY3	46424	46424	16(11)	0.1602
G. fascicularis DY4	44035	44035	17(12)	0.4725
G. fascicularis DY5	59792	59791	16(11)	0.2136
G. fascicularis DY6	50873	50873	17(11)	0.1698
G. fascicularis DY7	65241	65195	17(11)	0.1696

G. fascicularis DY8	62486	62380	14(11)	0.2026
G. fascicularis DY9	49368	48982	14(10)	0.1346
G. fascicularis DY10	59899	59719	13(9)	0.1429
P. lamellina SY1	66923	63316	13(10)	0.8407
P. lamellina SY2	68003	67866	17(12)	0.8487
P. lamellina SY3	158730	158549	20(12)	0.9959
P. lamellina SY4	50457	47829	18(13)	1.082
P. lamellina SY5	51552	50684	16(11)	1.19
P. lamellina XW1	59335	59171	16(11)	0.4452
P. lamellina XW2	78365	75907	18(12)	0.2901
P. lamellina XW3	69547	61510	17(11)	0.4796
P. lamellina XW4	72855	72851	18(12)	0.2886
P. lamellina XW5	63127	63122	17(12)	0.3152
P. *lamellina* DY1	48827	48815	12(10)	0.2219
P. lamellina DY2	54714	54711	16(11)	0.6017
P. lamellina DY3	52207	52193	15(11)	0.2632
P. lamellina DY4	44709	44624	13(9)	0.1555
P. lutea SY1	56417	56417	21(12)	1.382
P. lutea SY2	51987	51982	19(13)	1.42
P. lutea SY3	42551	42550	17(10)	1.287
P. lutea SY4	58271	58212	16(11)	1.363
P. lutea XW1	49838	49838	15(11)	1.421
P. lutea XW2	50811	50810	19(11)	1.419
P. lutea XW3	32753	32752	16(11)	1.425
P. lutea XW4	53971	53945	19(11)	1.455
P. lutea XW5	97876	97868	20(12)	1.42
P. lutea DY1	64584	64578	15(10)	1.399
P. lutea DY2	62205	62204	20(11)	1.4
P. lutea DY3	43176	43176	14(10)	1.409

P. lutea DY4	68522	68520	20(12)	1.381
P. lutea DY5	86645	86645	21(12)	1.406
S. glaucum SY1	69818	69787	15(12)	1.051
S. glaucum SY2	38865	38829	12(10)	1.032
S. glaucum SY3	60992	60962	12(10)	1.009
S. glaucum SY4	68007	67999	11(10)	1.019
S. glaucum XW1	53495	53225	13(9)	0.2097
S. glaucum XW2	44552	44424	15(11)	0.1821
S. glaucum XW3	67924	67863	13(10)	0.1884
S. glaucum XW4	52271	52222	17(12)	0.3419
S. glaucum XW5	59524	59381	18(11)	0.3351
Total(77)	4489717	4458888	32(13)	-
Seawater SY1	58132	6897	18(13)	-
Seawater XW1	7746	6885	18(12)	-
Seawater DY1	59523	6820	15(11)	-

Supplementary FIGURE 1. Rarefaction curves of *Symbiodinium* subclades in 77 coral samples from tropical SY, and subtropical XW and DY reef regions in the northern SCS. Each curve represents the observed number of *Symbiodinium* subclades in one coral sample.
