# Supplementary figures and images for: Flexible Symbiotic Associations of Symbiodinium With Five Typical Coral Species in Tropical and Subtropical Reef Regions of the Northern South China Sea
Source: Front Microbiol. 2018 Nov 2;9:2485. doi: 10.3389/fmicb.2018.02485 (PMC6225575; doi:10.3389/fmicb.2018.02485)

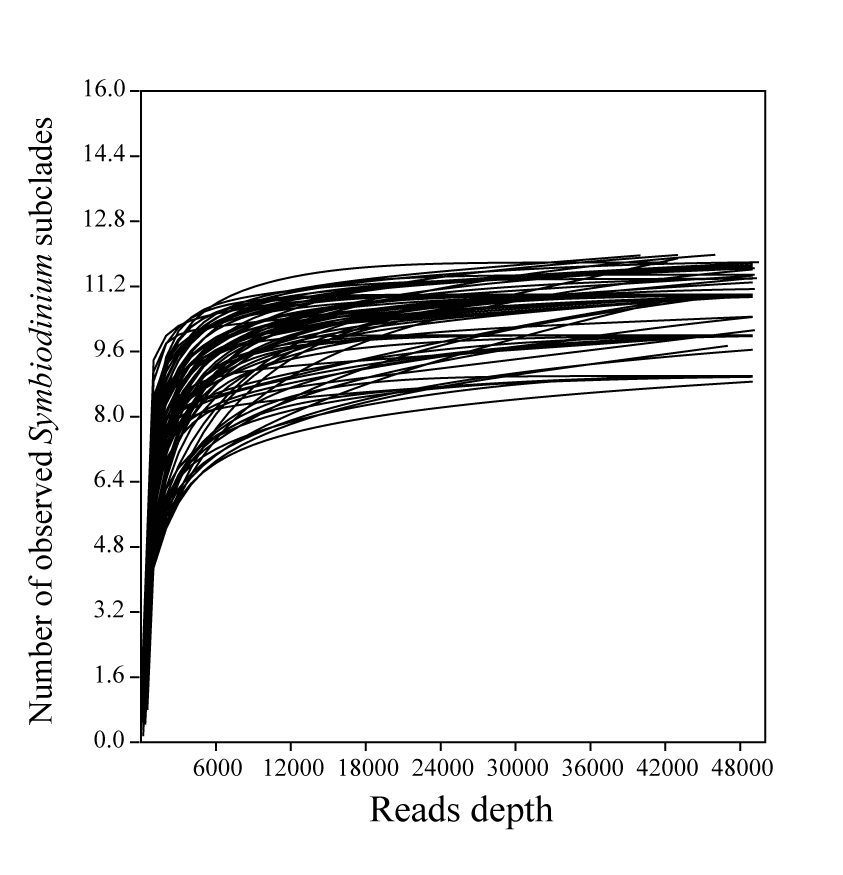

Supplement: Supplementary file 7 [file Image_1.TIF]
